# Use of radiofrequency ablation in the treatment of hepatocellular carcinoma: Experience of ablation protocols

**DOI:** 10.3892/etm.2012.706

**Published:** 2012-09-13

**Authors:** TAKAHASHI MASAYOSHI, NORITAKA WAKUI, YASUKIYO SUMINO

**Affiliations:** Division of Gastroenterology and Hepatology, Toho University Omori Medical Center, Tokyo 143-8541, Japan

**Keywords:** hepatocellular carcinoma, radiofrequency ablation

## Abstract

Hepatocellular carcinoma (HCC) is one of the most common cancer types worldwide. Percutaneous radiofrequency ablation (RFA) for HCC was introduced in Japan in 1999. It has been established as a major local treatment method worldwide including in Japan. On comparing outcomes between resection and RFA, they were comparable when cases were limited to those with 3 or fewer tumors of size 3 cm or smaller in many reports, based on which RFA has become the main treatment for small HCCs. RFA was introduced into our system at Toho University Medical Center Omori Hospital in 1999, and we treat nearly 200 HCC cases annually with RFA. Although individual medical facilities use their own methods of RFA, we would like to share our experience of RFA treatment protocols.

## Contents

1. Introduction
2. Differential use of needle electrodes depending on tumor diameter
3. Differential use of ablation methods with different needle electrodes
4. When expandable needle electrodes cannot be used due to tumor location
5. Selection of needle electrodes based on the condition of the surrounding hepatic tissue
6. Conclusion

## 1. Introduction

First reported by Buscarini *et al* in 1992 (1) and Rossi *et al* in 1993 (2), radiofrequency ablation (RFA) is an easy-to-operate and minimally invasive technique that provides effective local treatment. Subsequently, Shiina *et al* reported that, in cases with a small number (<3) of small (<3 cm in diameter) hepatocellular carcinomas (HCC), RFA was superior in terms of recurrence, and survival rates compared with the conventional HCC treatments of percutaneous ethanol injection therapy and surgical resection (3). As the rate of adverse events was similar among the three methods, they advocated the use of RFA as a first-line treatment for HCC (3). Chen *et al* also investigated the recurrence and survival rates of 180 patients with small HCC (<5 cm) who had been randomly assigned to either RFA treatment or surgical resection (4). They found no significant differences in these rates between the two patient groups and thus recommended RFA due to its minimal invasiveness.

RFA was introduced into our system at Toho University Medical Center Omori Hospital in 1999, and we treat nearly 200 HCC cases annually with RFA. Although individual medical facilities use their own methods of RFA, we would like here to share our experience of RFA treatment protocols.

## 2. Differential use of needle electrodes depending on tumor diameter

**Figure 1:**
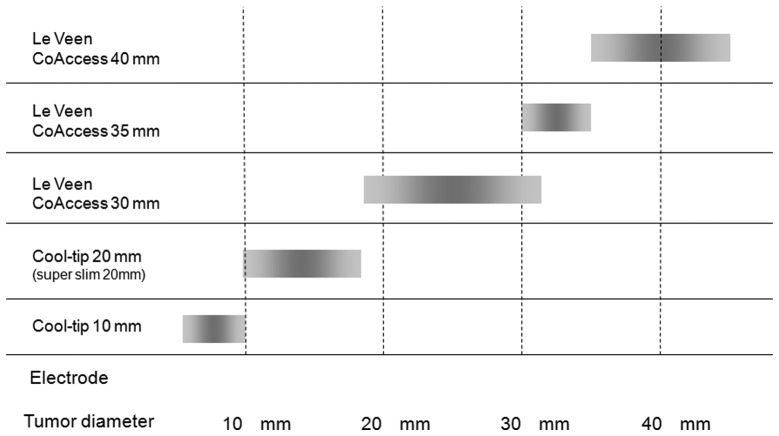
Tumor diameters and corresponding needle electrodes used in radiofrequency ablation (RFA) treatment.

Expandable LeVeen (Boston Scientific Corp., Natick, MA, USA) and monopolar Cool-tip (Covidien, Boulder, CO, USA) needle electrodes have been incorporated into our system since 1999 and 2002, respectively. Depending on tumor diameter, we perform RFA using either a LeVeen needle with an array diameter of 20, 30, 35, or 40 mm or a Cool-tip needle with a 10-mm or 20-mm non-insulated tip. Regardless of tumor size, all patients receive a single RFA treatment to avoid complications and dispersal of tumor cells due to repeated needle puncture. This protocol also offers the benefit of a shorter hospital stay. Bearing in mind the average ablative diameters afforded by individual needle electrodes and the minimal ablative margin of 5 mm or larger recommended by many studies (5), we investigated whether tumor size could serve as an indication for RFA. Based on the results, we now select needle electrodes according to tumor size (Fig. 1).

## 3. Differential use of ablation methods with different needle electrodes

### Cool-tip needle with a 20-mm tip

We previously used the liver of dead swine to show that the distance between the distal edge of ablation and the tip of a Cool-tip needle electrode was ≤2 mm (6). Accordingly, when no vasculature is present in the vicinity of HCC, we perform the following two-step ablation method to ensure a sufficient ablative margin from the needle tip and to avoid dispersal of tumor cells due to needle puncture.

**Figure 2:**
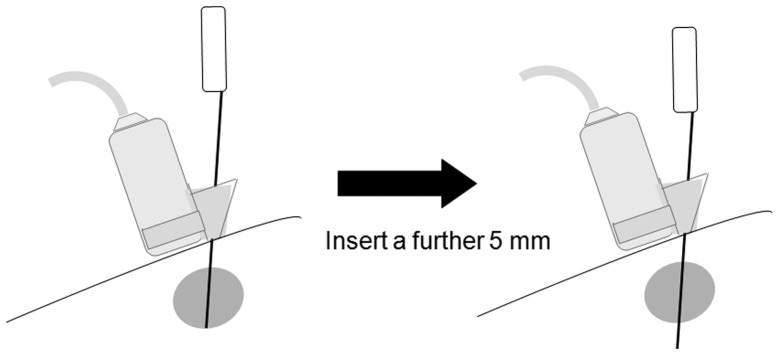
Illustration of the two-step ablation method. When no vessels are present near the hepatocellular carcinoma (HCC), the electrode is inserted into the base of the tumor, and 20–30 W of power is applied with two roll-offs. The electrode is then inserted a further 5 mm and 20–30 W of power is applied with one roll-off.

*Two-step ablation method (Fig. 2)*

- Insert the needle electrode into the tumor, hold the tip at the distal edge of the tumor, and turn the power on with the initial output set to 40 W.
- Increase the output by 10 W at 1-min intervals until reaching 60 W. When power roll-off has occurred twice while maintaining the output at 60 W, turn the power off.
- Insert the electrode a further 5 mm, repeat the above steps starting from 40 W, and end the treatment after achieving a single roll-off at 60 W.

**Figure 3:**
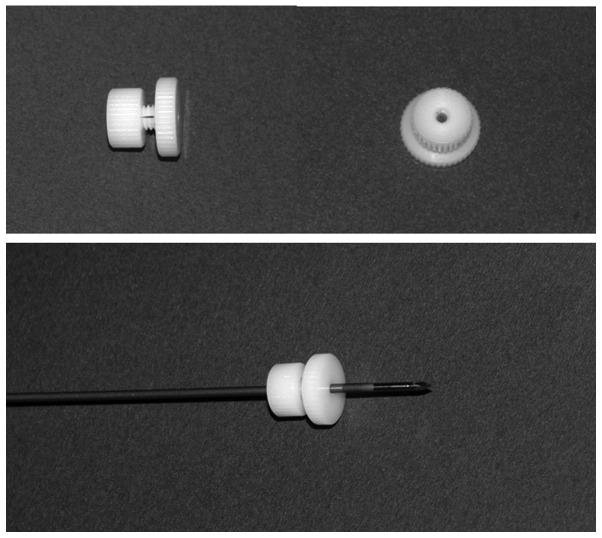
A photograph of the Duracon resin stopper for the percutaneous microwave coagulation therapy. The electrode is passed through the center of the stopper and the stopper is secured to the electrode by tightening the screw.

An ablative margin of 5 mm or larger will be ensured by advancing the tip further. A needle stopper made of Duracon for percutaneous microwave coagulation therapy is used to ensure accurate needle advancement (Fig. 3) (7). We perform RFA at low power, starting from 40 W up to a maximum of 60 W, because, unlike high-power ablation methods (8), this method prevents complications and does not affect the rate of local recurrence.

*Fixed ablation method.* If vasculature is present in the vicinity of HCC, inserting a Cool-tip needle down to the distal edge of tumor may result in vessel perforation. In such cases we perform a different ablation method as follows:

- Hold the tip of a needle electrode at the distal edge of tumor and turn the power on with the initial output set at 40 W.
- Increase the output by 10 W at 1-min intervals until reaching 60 W. When power roll-off has occurred three times while maintaining the output at 60 W, end the treatment.

### Cool-tip needle with a 10-mm tip

A Cool-tip needle with a 10-mm non-insulated tip is suitable for treating tumors with a diameter of <10 mm. It is particularly useful in cases with reduced hepatic functional reserve (7,9).

- Hold the tip of a needle electrode at the distal edge of the tumor and turn the power on with the initial output at 20 W.
- Increase the output by 10 W at 1-min intervals until reaching 30 W. When roll-off has occurred twice while maintaining the output at 30 W, turn the power off.
- Insert the electrode 5 mm further, repeat the above steps starting from 20 W, and end the treatment after achieving a single roll-off at 30 W.

(However, when vasculature is present in the vicinity of HCC, perform the stationary ablation method described above with the output starting at 20 W and perform treatment while maintaining the output at 30 W).

### LeVeen needle (Fig. 4)

With LeVeen needles, we perform the following steps to avoid the dispersal of tumor cells:

- Hold the tip of a needle electrode at the distal edge of the tumor, partially deploy the tines, and apply the power at the output initially set for the array diameter.
- Increase the output by 10 W at 1-min intervals until the power drops by 2 W, at which time maintain that output until roll-off occurs.
- Depending on the diameter of tumor, deploy the tines in a stepwise fashion (in 2–4 steps) and apply the power in the same way until the tines are fully deployed.
- Continue to apply 70% of the maximum output at the distal edge of tumor.
- Depending on the diameter of tumor, retract the electrode by approximately 5–10 mm, perform the same stepwise ablation with full deployment, and end the treatment.

## 4. When expandable needle electrodes cannot be used due to tumor location

When performing RFA of HCC in contact with a large blood vessel (hepatic vein, hepatic portal vein) just below the diaphragm and protruding from the distal surface of the liver, even for tumor >20 mm in diameter, an expandable LeVeen needle may perforate nearby vasculature of penetrate through the liver. In the case of HCC >20 mm in diameter, our conventional method of RFA using a Cool-tip needle with a 20-mm tip does not ensure a sufficient ablative margin, and a similar problem has been reported with the 30-mm tip (10). Therefore, for HCC >20 mm in diameter, we divide a tumor equally into three segments parallel to the direction of needle puncture. We then perform two-step ablation of the segment on the right by inserting a needle electrode into the distal end of the segment. The same steps are repeated with the segment on the left. This ensures an ablation of a wide area (11) and is useful in cases where the use of a LeVeen needle is not recommended, such as those mentioned above.

## 5. Selection of needle electrodes based on the condition of the surrounding hepatic tissue

**Figure 5:**
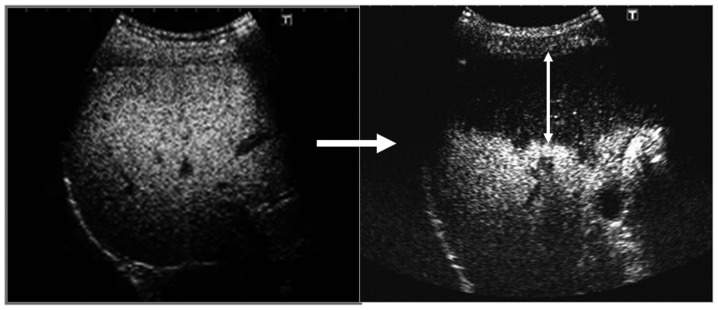
Measurement of microbubble collapse in the post-vascular phase of Sonazoid-enhanced ultrasonography 10 min after contrast infusion. The focus point was 6 cm. Flash-replenishment sequence settings included a mechanical index of 1.6 and number of beam transmissions set at 30. Then, we measured the depth of microbubble collapse from the liver surface after destroying the microbubbles in the scan volume with the use of high-transmission power ultrasound.

**Figure 6:**
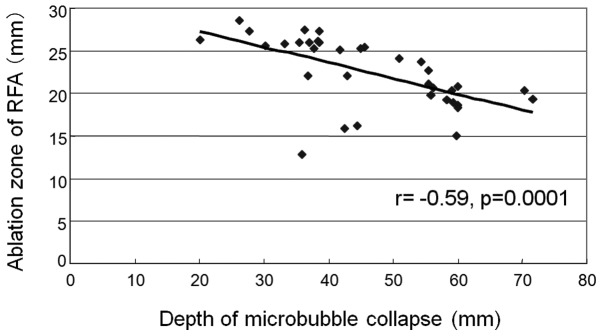
The association between depth of microbubble collapse and ablative zone diameters.

In some cases, RFA produces an unexpectedly small ablative margin. We previously reported that individual differences in ablative zone dimensions can be predicted by the depth of microbubble collapse in the liver parenchyma in the Kupffer phase of Sonazoid-enhanced ultrasonography (Fig. 5) (12). When an ablative zone is anticipated to be small because ultrasonography findings show a large area of microbubble collapse (Fig. 6), we select an electrode that produces a wider area of ablation than what we normally require to ensure a sufficient ablative margin.

## 6. Conclusion

We described the ‘tips and tricks’ of RFA treatment that we currently practice. It is our sincere hope that reporting our experience with RFA protocols will promote safe and highly effective performance of RFA treatment at all medical facilities.

## Figures and Tables

**Figure 4:**
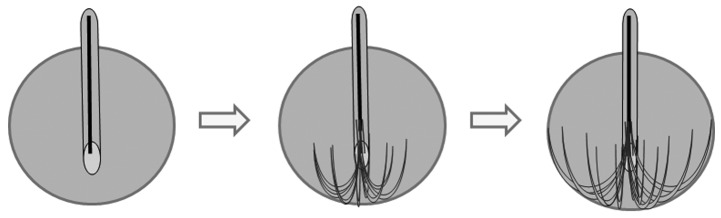
A stepwise ablation approach using a LeVeen needle.
